# Shape programming of polymeric based electrothermal actuator (ETA) via artificially induced stress relaxation

**DOI:** 10.1038/s41598-019-47949-0

**Published:** 2019-08-07

**Authors:** Yu-Chen Sun, Benjamin D. Leaker, Ji Eun Lee, Ryan Nam, Hani E. Naguib

**Affiliations:** 1 Department of Mechanical and Industrial Engineering, University of Toronto, Toronto, Canada; 2 Department of Materials Science and Engineering, University of Toronto, Toronto, Canada; 3 Institute of Biomaterials and Biomedical Engineering, University of Toronto, Toronto, Canada

**Keywords:** Mechanical engineering, Polymers

## Abstract

Electrothermal actuators (ETAs) are a new generation of active materials that can produce different motions from thermal expansion induced by Joule heating. It is well-known that the degree of deformation is determined by the amount of Joule heating and the coefficient of thermal expansion (CTE) of the material. Previous works on polymeric ETAs are strongly focused on increasing electrical conductivity by utilizing super-aligned carbon nanotube (CNT) sheets. This allows greater deformation for the same drive voltage. Despite these accomplishments with low-voltage actuation, many of the ETAs were constructed to have basic geometries such as a simple cantilever shape. In this paper, it was discovered that shape of polymeric ETA can be programmed into a desired configuration by applying an induced stress relaxation mechanism and post secondary curing. By utilizing such effects, an ETA can be programmed into a curled resting state which allows the actuator to achieve an active bending angle over 540°, a value far greater than any previous studies. This shape programming feature also allows for tailoring the actuator configuration to a specific application. This is demonstrated here by fabricating a small crawling soft robot similar to mimic an inchworm motion.

## Introduction

Over the past few decades there has been a tremendous amount of work devoted to the development of novel actuators that can respond to various stimuli such as light, heat, electric field, water, and compressed air^1–6^. Compared to traditional electric motors, these actuators offer advantages such as superior flexibility, better biomimicking ability, lower cost, and higher power-to-weight ratio. These properties make them more suitable for many applications such as lightweight prosthetics or soft robotics^7–10^. In addition, some of these actuators have even demonstrated performances that surpass biological muscles^11,12^. Electroactive polymers (EAPs) have the ability to undergo a large amount of strain or physical deformation when subjected to an external electric field. EAPs can be further classified into two subgroups: electronic and ionic. Due to the Coulomb force effect, electronic EAPs such as electrostatic and dielectric elastomers can achieve an actuation strain more than 100% in a short actuation time period; however, electronic EAPs are usually associated with an extremely high electrical field requirement that can be in the MV/m range^13,14^. On the other hand, ionic EAPs such as conducting polymers, carbon nanotubes (CNTs), and ionic polymer metal composites (IPMCs) require a lower voltage but an electrolyte environment is often needed for the ionic exchanges to take place^15–17^.

Silicon based thermal actuators were proposed in the late 1990s and early 2000s as a mean for accurate micro/nanoscale particle manipulation of *in situ* microscopy testing^18–21^. More recently, Chen *et al*. applied the same theory into a new type of EAP, polymeric based electrothermal actuator (ETA). This actuator utilizes Joule heating to drive the thermal expansion of a polymeric substrate^22^. In comparison to the EAPs reported previously, ETAs demonstrated a large deformation and low electrical field requirement. ETAs also have a fast response time (in the range of seconds) when compared to shape memory polymers but there is still progress to be made in order to be comparable with other smart materials such as dielectric elastomer (DEA) or IPMC. These materials can reach a response time in a milliseconds range^23^. Chen *et al*. dispersed 5 wt% of CNT into a polydimethylsiloxane (PDMS) matrix and demonstrated a 4.4% strain can be achieved under 52 V or 1.5 V/mm electric field^22^.

Later, the same group constructed a different ETA layout, by utilizing super-aligned CNT sheet to form an asymmetric bimorph layer structure with U-shape layout^24^. There are two major advantages to such a configuration: 1. enhanced electrical conductivity and heating distribution due to the CNT alignment, and 2. larger bending strain compare to uniformly dispersed CNT/PDMS composites due to the greater difference in the coefficient of thermal expansion (CTE) between the CNTs and the PDMS layer. As the CNT has a much lower CTE compared to PDMS, the differing degrees of expansion cause the actuator to curl towards the CNT side. Later research has shown that the actuation motion can be fine-tuned by adjusting the angle of CNT buckypaper alignment and cutting configuration, such as T or Z-shape^25^. Zeng *et al*. exchanged the PDMS matrix with CNT dispersed silicon rubber and waterborne polyurethane (WPU) and due to the large difference in CTE of the layers, activation can be achieved under 7 V^26^. Seo *et al*. showed that it is not necessary to use CNT buckypaper to create ETA^27^. Instead, the group spray-coated CNT solution on a glass slide and later applied a PDMS layer. This groups fabrication procedure allowed them to create a CNT coating on both faces of the actuator, allowing movement in two directions. With this configuration, the ETA can achieve a maximum bending angle close to 16° under 40 seconds.

Currently, there are very limited ETA studies that focus on enhancing the CTE of the bulk polymer via inserting secondary fillers as the resulting composites typically have a lower CTE than the original matrix^28^, not to mention that the incorporated secondary fillers usually have a lower CTE when compared to the primary polymer phase. Furthermore, it is very likely that the presence of fillers can also constrain the expansion of the polymer. On the other hand, recent research showed that the CTE can be controlled by utilizing volumetric expansion of liquid-to-gas phase transition. Zhou *et al*. improved the super-aligned CNT ETA reported in^24^ by increasing the number of CNT layers and further injected water into the actuator^29^. The “phase transition” ETA utilizes the expansion from both the PDMS and water vapor and the group demonstrated an extension more than 600% when under a driving voltage lower than 100 V. An alternative CTE enhancement approach was proposed Samel *et al*. by using expandable microspheres, which undergo a large amount of volumetric increase after heating up^30,31^. Instead to be used as an ETA, the initial designs proposed by the group can be implemented in microfluidic system as micro-valves or pumps when incorporated with an external heating source; however, the major disadvantage of the composite is that it can only provide one-time actuation due to the irreversible expansion of the microspheres. Nevertheless, an astonishing volumetric expansion over 270% can be achieved with this design. As a result, such one-time expansion actuators should not be overlooked as they may be useful for specific applications such as single-use smart bandages or fasteners.

As demonstrated in these studies, the initial or resting states of the ETA are always presented in a flat sheet configuration. One of the most recent ETA studies presented by Li *et al*. showed that different actuation motions can be achieved by utilizing the anisotropic property of the CNT sheet^32^. Nevertheless, such behaviour can not be viewed as true shape programming ability as the initial state is still in a 2D structure. In this study, a programmable CNT/PDMS ETA with variable initial actuation state is presented. It is reported that the CNT layer provides additional support to the ETA and shape programming can be achieved by utilizing the induced stress relaxation mechanism. This shape programming feature makes it possible to greatly improve the overall performance of the ETA as well as tailor the actuator to specific applications.

## Results and Discussions

### Material properties

As the motion of ETA is driven by Joule heating and thermal expansion, the ETA performance depends strongly on the material properties and can be described using the following relationships. Assuming linear thermal expansion, the changes in length (*ΔL/L*) of the actuator can be decreased with the CTE (α_L_) of the material and the change in temperature (*ΔT*):

1 $$\frac{{\rm{\Delta }}L}{L}={\alpha }_{L}{\rm{\Delta }}T$$

On the other hand, the change in the temperature of the material (ΔT = T_f_ − T_0_) depends on the change in its thermal energy (*ΔQ*) and the heat capacity of the material (*C*).

2 $${\rm{\Delta }}Q={\rm{\Delta }}TC=({T}_{f}-{T}_{0})C$$

To account for the radiation and convection heat loss, Equation (2) needs to be modified as the following:

3 $${\rm{\Delta }}Q=({T}_{f}-{T}_{0})C-{{\rm{\sigma }}}_{SB}\varepsilon {A}_{s}({T}_{f}^{4}-{T}_{0}^{4})-h{A}_{s}({T}_{f}-{T}_{0})$$

where *σ*_*SB*_ is the Stefan-Boltzmann constant, *ε* is the emissivity of the material, *A*_*s*_ is the surface area, and *h* is the heat transfer coefficient of the material.

Equation (3) can be rearranged into the following to isolate the change in the temperature of the material (ΔT = T_f_ − T_0_):

4 $${\rm{\Delta }}Q=({T}_{f}-{T}_{0})[C-{{\rm{\sigma }}}_{SB}\varepsilon {A}_{s}({{\rm{T}}}_{f}^{2}+{{\rm{T}}}_{0}^{2})({{\rm{T}}}_{f}+{{\rm{T}}}_{0})-h{A}_{s}]$$

5 $${\rm{\Delta }}T={T}_{f}-{T}_{0}=\frac{{\rm{\Delta }}Q}{C-{{\rm{\sigma }}}_{SB}\varepsilon {A}_{s}({{\rm{T}}}_{f}^{2}+{{\rm{T}}}_{0}^{2})({{\rm{T}}}_{f}+{{\rm{T}}}_{0})-h{A}_{s}}$$

In addition, the thermal energy (*ΔQ*) can be written in the form of power (*P*) and time (*t*) as expressed in Equation (6):

6 $${\rm{\Delta }}Q=P{\rm{\Delta }}t$$

Furthermore, the power (*P*) depends on both the input voltage (*V*) and the resistance (*R*) of the ETA:

7 $$P=\frac{{V}^{2}}{R}$$

By combining Equation (5) and Equation (7) into Equation (1), the change in changes in lengths (*ΔL/L*) can be written as:

8 $$\frac{{\rm{\Delta }}L}{L}={\alpha }_{L}\frac{{{\rm{V}}}^{2}{\rm{\Delta }}t}{R[C-{{\rm{\sigma }}}_{SB}\varepsilon {A}_{s}({{\rm{T}}}_{f}^{2}+{{\rm{T}}}_{0}^{2})({{\rm{T}}}_{f}+{{\rm{T}}}_{0})-h{A}_{s}]}$$

It can be expected that the radiation heat loss negated in comparison to the convection. As a result, the equation can be further simplified as:

9 $$\frac{{\rm{\Delta }}L}{L}={\alpha }_{L}\frac{{V}^{2}{\rm{\Delta }}t}{R[C-h{A}_{s}]}$$

It can be observed that in order to achieve maximum deformation, the actuator should have a high CTE and low electrical resistance. The actuation time *Δt* would depend on the rate of thermal distribution within the actuator; thus, material with higher thermal diffusivity would be more favored as such properties can shorten the response time.

Even though the elastomer used in this study is commercially available, the critical material parameters for an ETA, such as CTE, thermal diffusivity, and specific heat capacity, are not available. Basic characterization tests were first conducted and results are summarized in Table 1.

**Table 1:** Thermal properties of PDMS.

Study	CTE (μm/m∙°C)	Thermal Diffusivity (mm^2^/s)	Spec. Heat Capacity @ 200 °C (J/g °C)
Chen., *et al*.^24^	310	N/A	N/A
	6 (after embedded CNT)	N/A	N/A
This study	361.37 ± 13.44	0.1334 ± 0.013	3.023 ± 0.148

### ETA morphology

**Figure 1:**
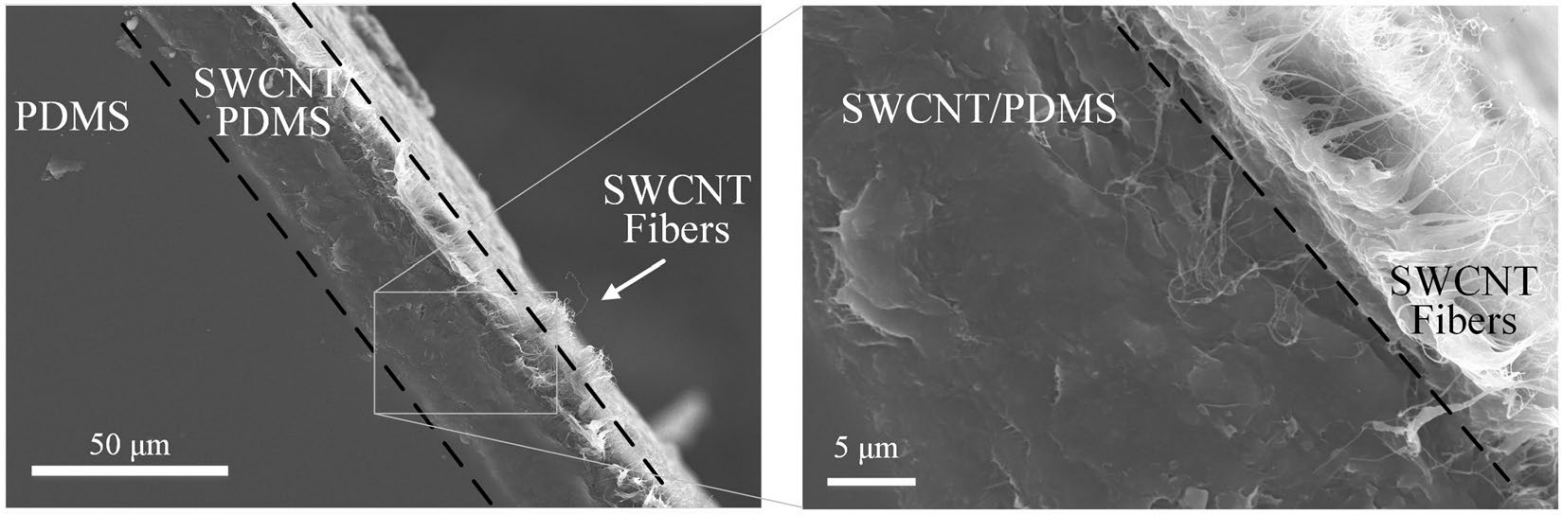
Cross sectional SEM images of fabricated SWCNT/PDMS ETA.

CNT/PDMS composites are well studied and many researchers focused on their compatibility and interaction areas^33,34^. The cross-sectional SEM images of the ETA are shown in Fig. 1. As the PDMS is directly casted onto the SWCNT buckypaper, 3 distinct layers can be observed: PDMS (left), PDMS/SWCNT interaction region (middle) and pure SWCNT layer (right). It can be observed that the PDMS elastomer matrix did not penetrate and surround the SWCNT layer completely but created a functionally graded layer. Starting from the left part of the image, the PDMS surface is smooth with almost no defects. When the uncured PDMS encounters the SWCNT layer, the uncured and free-flowing PDMS gel starts to penetrate the SWCNT layer and forms a rough SWCNT/PDMS interaction layer which is around 20 μm in thickness. Such region is critical as it determines the thermal energy distribution. To obtain optimized actuation results, the energy generated from Joule heating must conducted to the PDMS matrix though the SWCNT embed in the SWCNT/PDMS interaction region. As shown in the SEM image, the region ensured fast and uniform thermal distribution once Joule heating was initiated. In addition, the magnified SEM image shows that despite the PDMS infiltrating the majority of the SWCNT layer, the outer most layer of the SWCNTs buckypaper remains intact. As the SWCNT fibers are still observable, it is an indication that significant amount of Joule heating can be generated. Such a layer would be critical for providing the necessary conductivity for Joule heating of the ETA.

### Actuation of ETA

**Figure 2:**
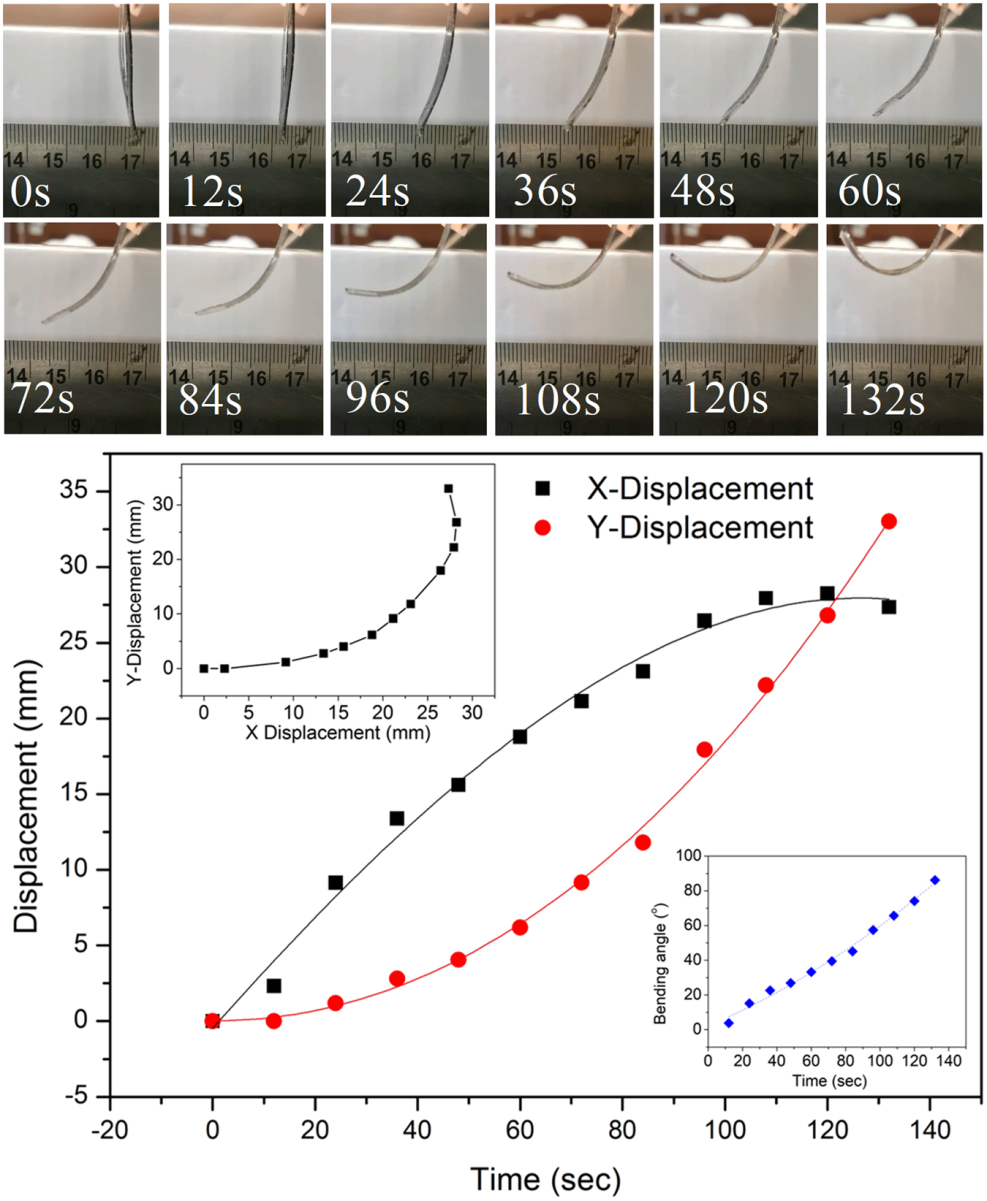
Actuation, tip displacement, and bending angle of PDMS ETA.

The top images in Fig. 2 show a time-lapse demonstration of the actuation of the PDMS ETA while the corresponding horizontal/vertical tip displacements and the bending angles are plotted in the bottom. The bending angles are calculated from measuring the displacement of the ETA tip in both x and y directions. The actuation video can be found in the supplementary document. As shown in the figure, the actuator has similar deformation behaviour when compared to ETAs reported previously: Seo *et al*. reported a maximum 3.3 mm displacement can be achieved under 60 V/48 mA steady state condition^27^ while Chen *et al*. reported a maximum 10 mm displacement under 20 V/40 mA supplied power^24^. Zeng *et al*. reported that the combination of 7 V and 0.97 A can produce a horizontal tip displacement close to 30 mm^26^. Since the horizontal displacement of the ETA is likely dependent on the length of the actuator (longer actuation arm can create more deflection), a valid displacement comparison would be normalizing the deflection with the movable length of the ETA. Applying this normalization, the ratio of maximum tip deflection to the cantilever length reported from previous studies demonstrated values of 0.12^27^, 0.32^24^, and 0.58^26^; horizontal displacement ratio calculated from this study is 0.82 and the vertical displacement ratio is 0.95, far larger than previously reported values, as summarized in Table 2. In addition to the greater deformation, the driving voltage for the PDMS ETA used in this study (12 V) was significantly lower than most literature, with the exception of 7 V voltage in^26^.

**Table 2:** ETA performance comparison chart.

Study	Material	Voltage (V)	Current (mA)	Displacement	Normalized Displacement (w/cantilever arm)
Chen *et al*.^24^	Aligned CNT paper/PDMS	20	40	Bending tip, 10 mm	0.32
Seo *et al*.^27^	SWCNT + SWCNT/PDMS	60	48	Bending tip, 3.5 mm	0.12
Zeng *et al*.^26^	MWCNT/PU/rubber/Cu rods	7	970	Bending tip, 30 mm	0.58
**This study**	**SWCNT** + **PDMS**	**12**	**650**	**X: 25** **mm Y: 33** **mm**	**X: 0**.**82 Y: 0**.**95**

In the literature, most studies do not report the vertical displacements as the values are usually less significant in comparison to the horizontal deformation. One exception is the study conducted by Li *et al*., who reported a maximum curvature angle close to 180° can be achieved under 25 V, 0.17 A^25^. The group further justified that such large angle was achieved because the ETA was constructed from super-aligned CNT sheets. On the other hand, the results shown in Fig. 2 indicate that ETA with randomly orientated CNT can also achieve similar performance to the results reported by Li *et al*. which was close to 90° bending or 180° degree of curvature^25^. This result suggests that ETA can undergo large deformation as long as high electrical conductivity is maintained.

**Figure 3:**
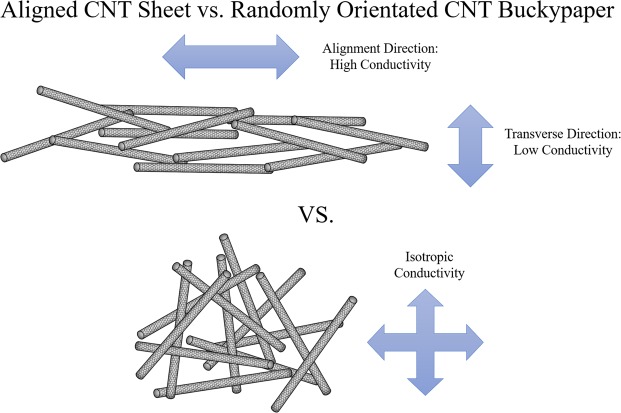
Comparison between aligned CNT and randomly distributed CNT.

When comparing the electrical conductivity between anisotropic and super-aligned CNT sheet with randomly distributed CNT buckypaper, anisotropic aligned CNT sheet would have a superior electrical conductivity in the direction parallel to the CNT alignment. However, such alignment also implied that interactions between individual CNTs would be less in the direction perpendicular to the CNT alignment (Fig. 3), as decrease in conductivity was reported^35^. Inoue *et al*. reported the sheet resistance in the parallel direction of MWCNT buckypaper as 13.8 Ω/sq, which is significantly lower than that in the perpendicular direction, 100.1 Ω/sq^35^. Li *et al*. utilized the anisotropic conductivity to produce a different desirable bending direction since the strong CNT/CNT interaction region can overcome the insufficient conductivity in the transverse direction^25^. In addition to the highly entangled CNT network, buckypaper also integrated well into the PDMS matrix, which implied a fast and uniform thermal energy transition can be achieved for activation. Lastly, the CNT utilized in this study is single-walled carbon nanotubes which has a higher surface area compared to mutli-walled carbon nanotubes utilized in literature. The increase in surface area contributed to the increase in thermal energy transfer efficiency, therefore our study indicates that randomly orientated SWCNT can achieve a similar actuation performance when compared to the ETA fabricated with a super-aligned CNT sheet.

### Shape programming of ETA

**Figure 4:**
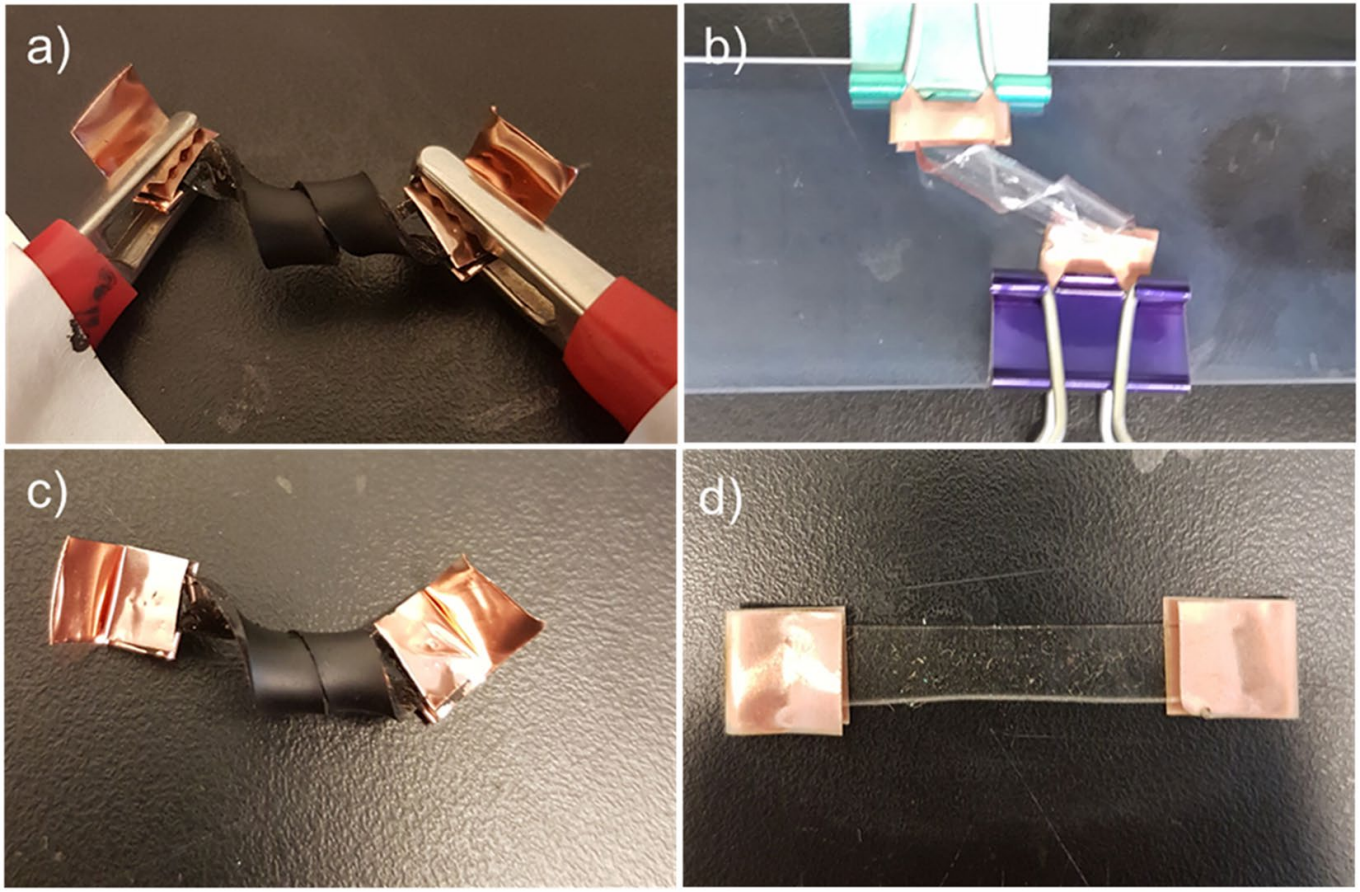
Shape programming demonstration of (**a**) SWCNT/PDMS ETA (**b**) pure PDMS and (**c**,**d**) after programming.

One of the most intriguing properties of this ETA design is the shape programming property which has not been reported in any previous study. Such property is extremely important for the application driven product development, as the shapes of ETA are no longer restricted to 2D sheets or pieces. The shape programming capability is demonstrated in Fig. 4. First, the thin film ETA was manually deformed into the desired curved shape (in this case, 720° spiral as shown in Fig. 4a). While maintained in this position, a low voltage (7 V) was applied for a prolonged period of time (1 hour). The voltage was then removed and the ETA was still held in the desired configuration while cooling. After removing the electrodes and wires, it was observed that the ETA did not revert to its pre-programmed shape (flat) but rather preserved the curved configuration (Fig. 4c). Further testing was conducted on pure PDMS sample and the results indicate that the SWCNT layer played an important role in the shape programming process. Since the pure PDMS has extremely high resistance, clamped samples were placed in a high temperature (200 °C) oven instead of Joule heating. As shown in Fig. 4b,d the PDMS sample immediately returned to its strip shape once the clamps were removed and no shape fixing can be observed.

The behaviour reported in this study is profoundly similar to a well-studied smart material: thermally induced shape memory polymers (SMPs). The SMP usually has two different phases: a thermally reversible phase and a shape fixing phase. In addition, the shape programming of SMP involves the following steps: (a) SMP is heated above the glass transition temperature (Tg) of the thermally reversible place; (b) physical deformation is applied to hold the SMP in its temporary shape; (c) temperature is lowered below the Tg and SMP is “frozen” in the temporary shape; (d) by increasing the temperature above the Tg, the SMP can revert its original orientation from the temporary shape^36–42^.

The ETA and SMP possess several similarities which can be summarized in Table S1. As it can be observed, both active materials need to be programmed at a higher temperature; the temporary shape is fixed by lowering the temperature; and lastly, re-heating initiates the actuation. Despite the similarity of the shape programming processes, they are operated under completely different mechanisms. The shape recovery of the SMP depends on the variation in the thermal behaviour of the two distinct polymer phases. On the other hand, the cross-linked PDMS ETA is composed of one single polymer and hence does not show any shape memory effect by itself, as shown in Fig. 4b,d. Thus, it can be concluded that the shape programming of ETA is not driven by the same thermal properties as SMP.

**Figure 5:**
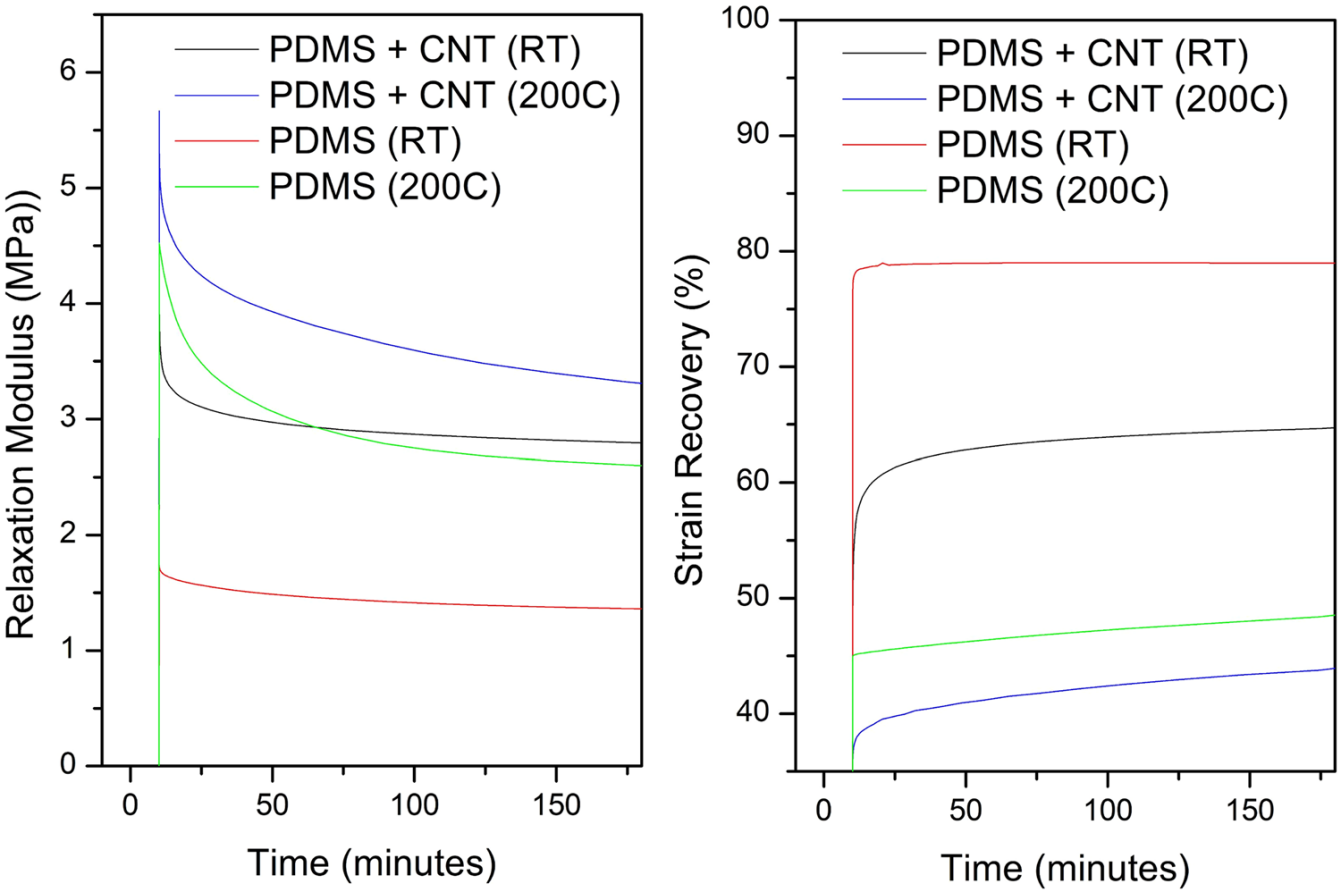
Stress relaxation (left) and the following strain recovery (right) of pure PDMS and PDMS + CNT composites under different condition.

It is proposed that stress relaxation behaviours can be used to better explain the shape programming effect of this ETA. To confirm the hypothesis, ETA composites and pure PDMS were tested using a DMA under 25 °C (RT)/200 °C environment and the results are shown in Fig. 5. The initial 5% stretching is reflected as the initial spike in relaxation modulus: samples containing the CNT layer have higher relaxation modulus due to the stronger mechanical properties, as expected. In addition, tests conducted at 200 °C shows higher modulus than room temperature tests for both pure PDMS and PDMS + CNT samples. Previous study has shown that temperature has a significant effect on mechanical properties over long periods of time^43^. One possible explanation for this result is that the high temperature environment increased the degree of crosslinking within the PDMS. Pure PDMS under room temperature conduction show a poor shape fixing ability (80% strain recovery), as expected. It can be inferred from the missing 20% recovery that stress relaxation had already taken place as the PDMS had yet to reach its plastic deformation region with 5% applied strain^43^. With the CNT layer, however, strain recovery dropped to 65% as the high stiffness CNT restrained the PDMS from recovering to its original shape. At higher temperature, both pure PDMS and PDMS/CNT samples exhibit a further decrease in the strain recovery which is likely due to an increase in the degree of crosslinking. Such results also echo with the results shown in Fig. 4c,d of the helix shape ETA and the non-programmable pure PDMS, respectively.

In Fig. 4c, helix shape ETA was able to retain its configuration after heating due to the programming effect while the pure PDMS sample failed to hold its programmed position despite a prolonged heated condition. Furthermore, the 200 °C DMA test was in fact under a much higher temperature compared to the internal temperature of the CNT ETA during programming (<150 °C). The purpose of the DMA experiment is to verify that even under extreme temperature conditions, pure PDMS does not have the ability to retain its shape, which also backs up the theory of relaxation and echoes with the results shown in Fig. 4. Further characterization of the possible PDMS post-curing effect can be found in the supplementary document. Consequently, it can be concluded that the Joule heating can enhance both the stress relaxation and degree of crosslinks, thus resulting the shape programming ability for the SWCNT/PDMS ETA.

### PDMS post-curing and thermo-mechanical verification

**Figure 6:**
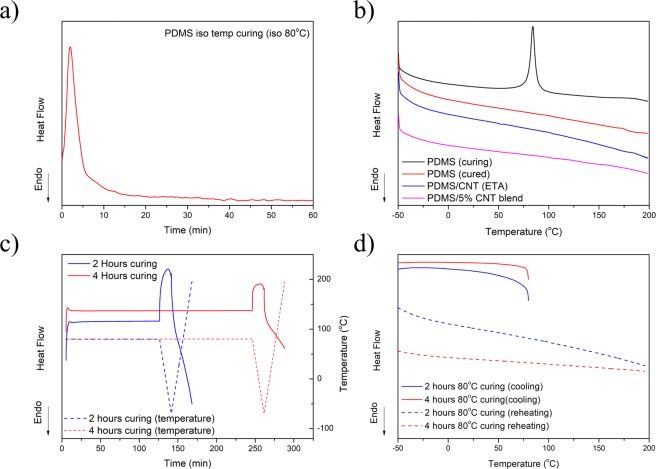
(**a**) Isothermal of PDMS curing behaviour, (**b**) DSC temperature ramp of different PDMS samples, (**c**) 2 & 4 hours 80 °C curing behaviour with respect to time, and (**d**) 2 & 4 hours 80 °C curing behaviour with respect to temperature.

To further study the possible post-curing effect of PDMS contributing to the shape programming behaviour, DSC experiments were conducted. To simulate the curing procedure, uncured PDMS was subjected to an isothermal testing condition at 80 °C, as shown in Fig. 6a. It can be observed that the majority of the curing was completed during the first 10 minute under isothermal condition. Such result reveals that the 2 hours under 80 °C curing time for sample fabrication was sufficient. According to literature, PDMS has a Tg close to −125 °C^44–48^ while incorporation of nanoparticles has either little or no effect on the Tg. For example, Panou *et al*. have shown that Tg maintained a constant value at −123 °C when 5, 10, 15 wt% polyethylene glycol (PEG) is blend with the PDMS^44^. Sepúlveda *et al*. reported no observable changes of Tg with 1 volume fraction of aligned CNT within PDMS as such amount is not sufficient to influence the polymer chain mobility^49^. Fragiadakis *et al*. reported a maximum 3 °C variation in Tg when 6, 9, 10, 16 volume percent of silica nanoparticle incorporation^50^. As the ETA fabricated from our study has distinct buckypaper and ETA layer, the Tg of the SWCNT/PDMS ETA should be close to the Tg of the pristine PDMS.

Figure 6b is the temperature ramp results of the uncured and cured ETA. It can be observed from the figure uncured PDMS started to cure at a temperature above 70 °C and reaching maximum peak around 80 °C, as expected. No exothermal exchange peak can be observed for both the cured PDMS and SWCNT/PDMS ETA samples, which indicates that the samples were fully cured. To further justify the DSC result, we further fabricated a SWCNT/PDMS blend that containing 5 wt% SWCNT and no difference in thermal history can be observed. To study the curing time effect on the PDMS, uncured PDMS was heated from room temperature to 80 °C and held for 2 and 4 hours. It can be observed from Fig. 6c, no thermal exchange can be observed during the 80 °C isothermal condition, indicating that no post curing took place. Figure 6d, temperature ramping result shows similar behaviour to Fig. 6b, of which suggested that curing condition is not time dependent.

**Figure 7:**
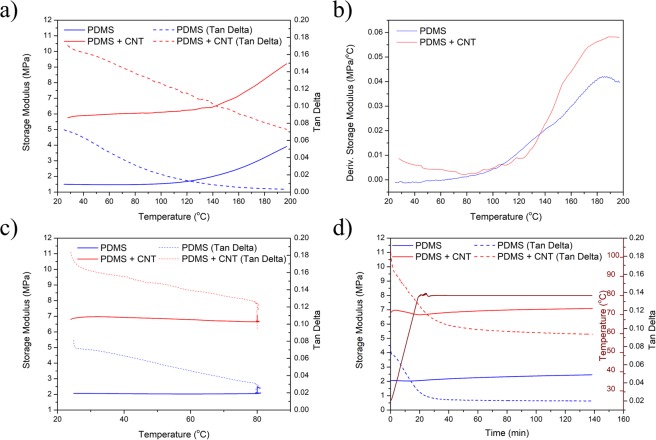
(**a**) Storage and loss modulus of PDMS & PDMS + CNT ETA under temperature ramping, (**b**) derivative of the storage modulus, (**c**) heat-and-hold experiment with respect to temperature, and (**d**) heat-and-hold experiment with respect to time.

To study the thermal-mechanical behaviour of the ETA, further DMA temperature ramp tests on both PDMS and PDMS/CNT ETA samples were conducted. Figure 7a shows the storage and loss modules of pristine PDMS and ETA. It can be expected that both storage and loss modulus of ETA would be higher than the PDMS due to the presence of high stiffness SWCNT buckypaper. It can also be observed that storage modulus of both samples started to increase with temperature. When taken the derivate (Fig. 7b) it can observe that the ETA sample has a more dramatic increase in the storage modulus, which can be correlated to the shape programmability of the ETA shown in this study. Such result further supports that the CNT buckypaper is necessary for achieve the shape programmability. Figure 7c,d shows the effect of different curing time on pristine PDMS and ETA samples. It can be observed that storage modulus maintained the same value under an additional 2 hours of 80 °C curing, as the presence of a CNT layer played a more significant role in the shape programmability. However, PDMS post curing behaviour cannot be identified from both DSC and DMA analysis.

### ETA applications with different programmed shape

**Figure 8:**
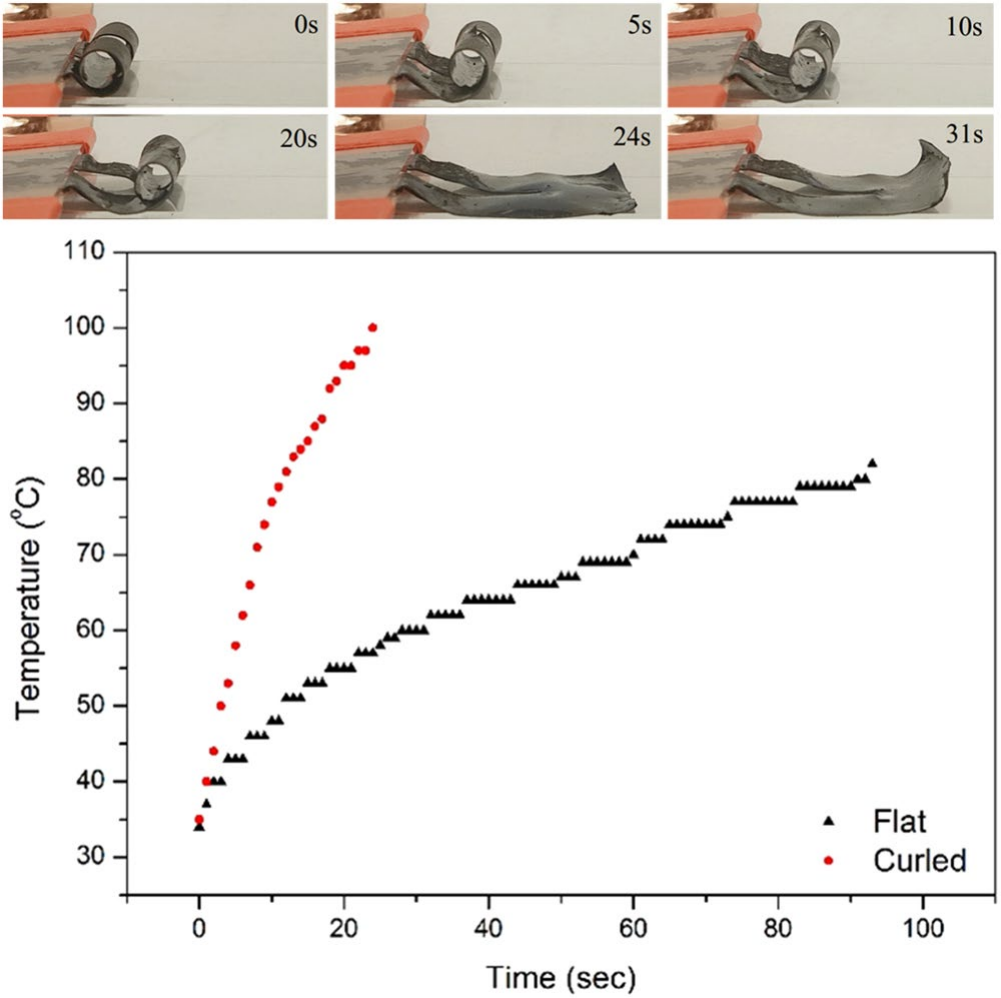
ETA actuation from a programmed curled shape and temperature changes compared to non-programmed (flat) configuration.

By utilizing the shape programming ability of the ETA, it is possible to achieve novel ETA configurations that improve the overall actuator performance. This is demonstrated here with a programmed curled resting state ETA (Fig. 8). Such state is given the name “programmed shape”, which is also comparable to the “temporary shape” in SMP. Under 7.8 W (12 V) the actuator curled around itself 1.5 times, equivalent to a bending angle of 540°. This is far larger than any bending angle reported previously, including ETAs fabricated from super-aligned CNT buckypaper^22,24–27^. In addition, the 12 V driving voltage was also lower than the literature, except the 7 V used in^26^. There are mainly two reasons behind the large deformation of ETA: the fast heating rate and randomly aligned CNT configuration. Also shown in Fig. 5, the curved ETA had a much faster heating rate. When the ETA was programmed into the curled state, it could reach 100 °C in less than 30 seconds. On the other hand, the pre-programmed (thin film) ETA only reached 80 °C after 90 seconds, significantly longer than the programmed state. It is possible that the heat loss of the curled ETA was less during the activation as there was less surface area exposed to the environment. As a result, the curled ETA had a faster initial heating and a higher maximum temperature. Furthermore, it is possible that the randomly aligned CNT buckypaper also contributed to the large bending angle, due to the lower stiffness in comparison to the super aligned buckypaper^51^. Furthermore, Li *et al*. demonstrated that the CNT alignment can have significant impact on the bending direction of the ETA^25^. As a result, the isotropic mechanical property of a lower stiffness buckypaper would have less mechanical restraints compared to super-aligned CNT buckypaper, thus making larger bending angle possible.

In addition to the large deformation, the actuation speed was also extremely fast: a full opening of the curved state can be achieved under 25 seconds. The fast actuation speed was the result of faster heating rate, as shown in Fig. 8. It can be expected that the CNTs within the conductive layer were more tightly packed when the ETA was in the curled shape, therefore, Joule heating was more effective and resulted in both faster heating rate and actuation speed. It can also be noted that there’s a burst of uncurling action between 20 and 24 seconds. It is possible that the uncurling is due to the elastic energy stored at the very tip of the actuator. As the voltage was applied, the actuator expanded as much as possible; however, the tip was being constrained by the configuration. Once the ETA uncurled enough so that the tip was free (20 seconds), it could suddenly flatten out, as shown in the image at 24 seconds. After the ETA was completely uncurled, the amount of heat loss was increased, thus it had the tendency to recover to its curled programmed shape at 31 seconds. After the voltage was turned off, the ETA stayed in the configuration shown at 31 seconds. It should be noted that such configuration is not the secondary programmed shape but it is the partial recovery of the ETA attempting to recover back to its programmed curled configuration.

Even though fast actuation motion and large bending angle can be achieved, such design also exhibited some significant drawbacks to be address in future work. It was observed that after actuation, the ETA was not able to recover to its initial state. It is possible that the high heat generated during the actuation started to override the programming of the curled state, therefore the ETA was not able to fully recover its original configuration.

**Figure 9:**
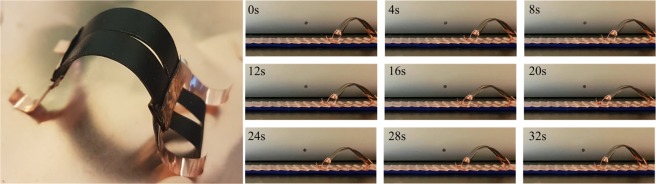
ETA crawling soft robot and the actuation behaviour (video available in supplementary document).

Another use of the shape programming ability is to create actuators tailored to a specific application. This allows for creative implementations of the ETA and is demonstrated here through a small crawling soft robot (Fig. 9). Shape programming was used to set a rectangular ETA strip into a slightly curved resting state, with the CNT layer on the outside of the curve. As a result, an applied voltage causes the actuator to flatten out. This design allows the actuator to mimic a worm under a cyclic voltage. 12 V was supplied to the actuator from two metallic rails with a 20 seconds ON-OFF cycling time. The ETA started to expand once the voltage is ON, from 0 to 20 seconds. After the voltage was switched off, the ETA would return to its curved state due to the fast heat dissipation. The ETA is allowed to move forward due to the ridged rail design. In contrast to the curled resting state ETA shown in Fig. 8, the crawling robot shown in Fig. 9 was programmed into a much smaller curvature. Therefore, the crawling robot was able to move forward by cycling between an expanded and a contracted state in response to the voltage signal. On the other hand, the ETA shown in Fig. 8 was not able to fully recover to its curled configuration as the ETA was programmed into an extremely large curvature. This leads to the only observable partial recovery. The video of the crawling robot can be found in the supplementary document.

## Conclusions

This study presented an ETA using randomly oriented CNTs that was capable of significant deformation with low drive voltage in a standard cantilever configuration. This ETA also possessed a shape programming ability due to induced stress relaxation. It was shown that the stress relaxation is only applicable with the SWCNT layer, as pure PDMS does not show the same programmability. By utilizing this effect, it was shown that the ETA can be programmed into a curled resting state that is capable of a bending angle over 540° under a 12 V drive voltage. In addition, a small crawling soft robot was fabricated by applying the same concept. This work presented novel features for ETAs, the shape programming ability, which will help to develop the next generation of ETAs and push them closer to real world implementation.

## Methods

### Fabrication of dual layer ETA

For fabricating the ETA, a layer of conductive single-walled carbon nanotubes (SWCNT, Tuball, >75% purity, obtained from OCSiAl, Luxembourg) was first prepared. The SWCNT buckypaper was prepared by solvent casting/evaporation method. A SWCNT/acetone solution with a concentration of 0.83 mg/mL was prepared by magnetic stirring enhanced sonication with 4 seconds pulse-on and 2 seconds pulse-off setting over a total duration of 67.5 minutes. The dispersed solution was poured into a glass petri dish and the solvent was evaporated under a room temperature environment. The resulting SWCNT film was highly flexible and maintained high structural integrity after deformation, as shown in Supplementary Fig. S1. The resultant film weighed approximately 0.025 g with a resistance of 6.7 ± 1.2 Ω when measured across the 6 cm diameter disc.

A commercially available silicone based rubber elastomers materials, PDMS (Sylgard 184, supplied from Dow Corning) were used in this study. Sylgard 184 was prepared from blending curing agent to the uncured matrix with a mixing ratio of 10:1 The uncured polymers are then poured on top of the SWCNT layer. All samples were subjected to degassing processes to remove trapped air bubbles during the resin/crosslinker mixing step. Degassed samples were then transferred to 80 °C for 2 hours to cure. After curing, samples can be cut into the desired shape. Copper sheets were clamped to the end of the actuator to be used as electrodes and a small amount of carbon ink was further applied to the electrode/ETA junctions to ensure proper electrical contact.

### Characterization

As the expansion of elastomer plays a significant role in the overall ETA performance, a series of material properties were tested. Scanning electron microscopy (SEM, JSM-IT100, JOEL Inc.) was used for studying the SWCNT/elastomer interface region. CTE was measured with a thermomechanical analyzer (TMA, Q400, TA Instrument) at a 5 °C/minute heating rate from room temperature to 200 °C. Thermal diffusivity was measured with a thermal constants analyzer (TPS2500S, ThermTest Inc.). Heat capacity was measured with a differential scanning calorimetry (DSC, Q2000, TA Instrument). The stress relaxation behaviour was studied by using a dynamic mechanical analyzer (DMA, Q800, TA Instrument) with a tensile fixture. Samples were first stabilized in either a 25 °C or 200 °C environment for 10 minutes. A 5% tensile strain was applied for 3 hours and the relaxation stress was monitored. After 3 hours, the applied stress was released and the strain recovery was recorded over a 2-hour period.

Post-curing of PDMS behaviour was conducted by using differential scanning calorimetry (DSC, Q2000, TA Instrument). To simulate the curing procedure, uncured PDMS was subjected to an isothermal testing condition at 80 °C while temperature ramping testing was conducted from −50 °C to 200 °C with a 10 °C/min heating rate. To study the possible time-dependent curing effect, uncured PDMS was heated to 80 °C with a 10 °C/min heating rate and left isothermal for 2 and 4 hours, respectively. The cured sample then cooled to −50 °C and was reheated to 200 °C with the same 10 °C/min heating rate to capture any possible thermal exchange of uncured PDMS resin.

To further study the thermal-mechanical response, DMA tests were also conducted in under temperature ramping condition from room temperature to 80 °C with a heating rate of 3 °C/min heating rate. To study the effects of various curing rates, both the PDMS and SWCNT/PDMS ETA samples were subjected to an isothermal condition of 80 °C for an additional 2 hours.

For measuring the deformation and Joule heating of the ETA actuator, a U-shape actuator was used (dimension of the actuator can be found in supplementary, Fig. S2). A thermocouple was placed in the middle of one of the legs of the U-shape ETA and in contact with the elastomer side of the actuator with a sampling rate of 1 Hz. The displacement was taken as the maximum deflection at the tip of the actuator.

## Supplementary information

PDMS Cantilever x32 speed

crawling ETA x32 speed

supplementary
