# Supplementary material for: Shape programming of polymeric based electrothermal actuator (ETA) via artificially induced stress relaxation
Source: Sci Rep. 2019 Aug 7;9:11445. doi: 10.1038/s41598-019-47949-0 (PMC6685997; doi:10.1038/s41598-019-47949-0)
Supplement: Supplementary file 3 — supplementary [file 41598_2019_47949_MOESM3_ESM.docx]

**TITLE:** " Shape programming of polymeric based electrothermal actuator (ETA) via artificially induced stress relaxation "

Yu-Chen Sun ^a, b, c^, Benjamin D. Leaker ^a, b, c^, Ji Eun Lee ^a, b, c^, Ryan Nam ^a, b, c^, and Hani E. Naguib ^a, b, c^*

^a^ Department of Mechanical and Industrial Engineering, University of Toronto, Toronto, Canada

^b^ Department of Materials Science and Engineering, University of Toronto, Toronto, Canada

^c^ Institute of Biomaterials and Biomedical Engineering, University of Toronto, Toronto, Canada

5 Kings College Rd., Toronto, Ontario, Canada, M5S3G8

Contact email: [naguib@mie.utoronto.ca](mailto:naguib@mie.utoronto.ca)

**Supplementary Document**

*Table S1 – Programming and actuation behaviour comparison of ETA and SMP*

| ***Actuator type*** | ***Programming process*** |
| --- | --- |
| *Electrothermal Actuator* | - *The initial configuration of a newly fabricated ETA is a flat/thin film (pre-programmed shape).* - *Low Joule heating (7V) for shape programming results in a curved (programmed or temporary shape) configuration, as shown in Figure 5 @ 0s.* - *High Joule heating (12V) for actuation results in the shape recovery from the programmed shape to the pre-programmed shape, as shown in Figure 5 @ 24s.* - *Voltage off results in the partial recovery of the ETA, as shown in Figure 5 @ 31s.* |
| *Shape Memory Polymer* | - *The initial configuration of a newly fabricated SMP is any desired pre-programmed or permanent shape.* - *Loading or deformation can be applied under the condition of T > T_g_ which results in a programmed or temporary shape* - *When temperature is lowered to T < T_g_, the programmed shape is maintained after removing the applied load.* - *When temperature is increased to T > T_g_ again, the pre-programmed or permanent shape is recovered.* |


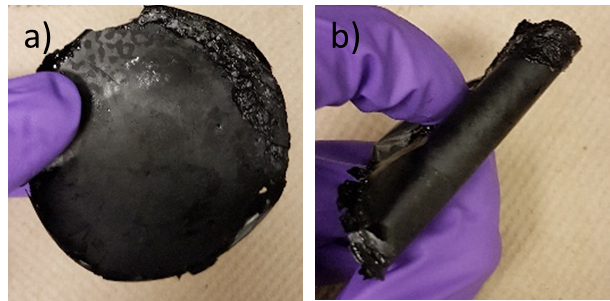


***Figure S1*** *– (a)SWCNT film fabricated from solvent evaporation, and (b) flexing of SWCNT film*


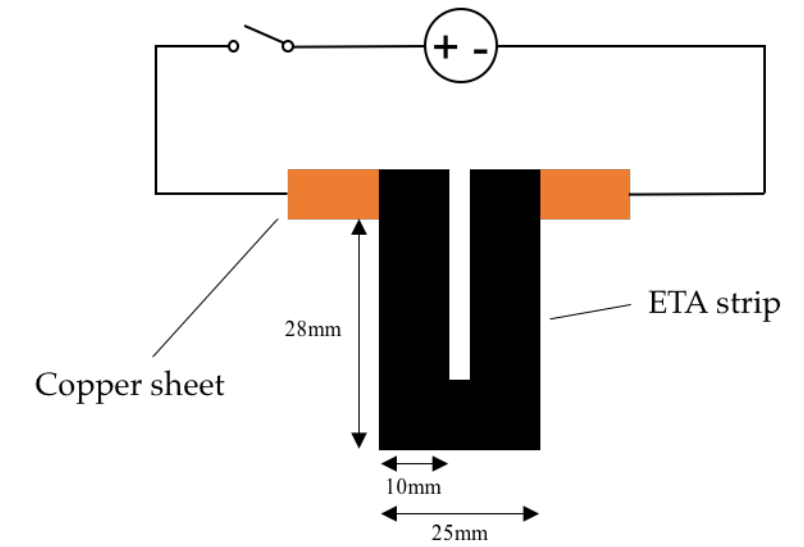


**Figure S2** - Dimension of U-shape actuator


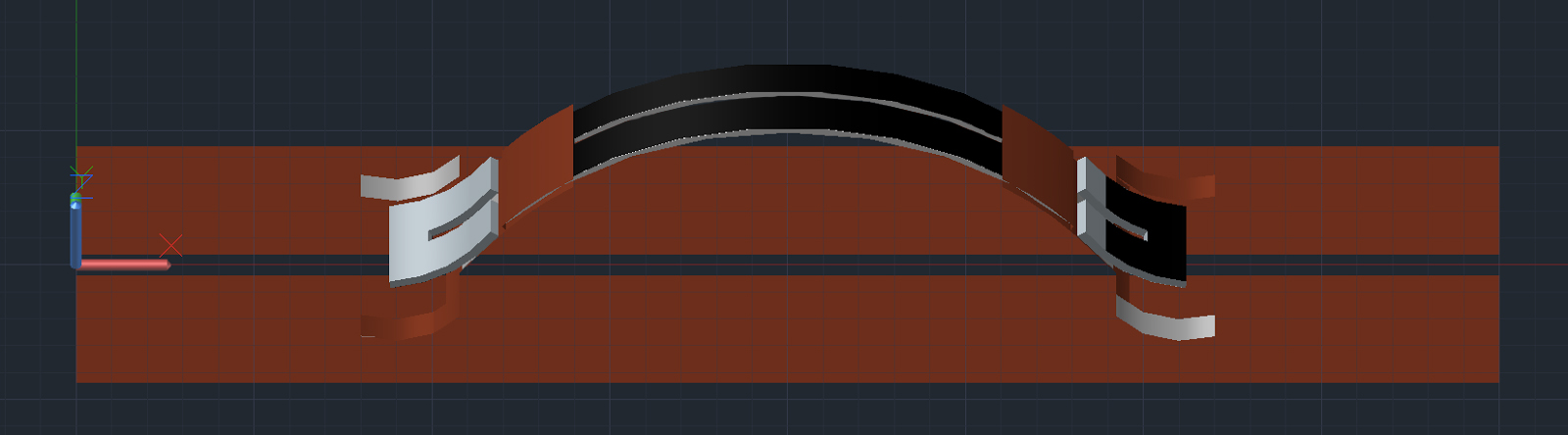


**Figure S3 –** CAD model of ETA crawling soft robot

**PDMS NMR characterization**

To further investigate into whether the post crosslinking is contributed to shape programming effect, NMR experiment was conducted. PDMS was first cured in an 80^o^C oven for 2 hours, similar to the ETA fabrication methods. Once cured, the PDMS film was placed in a sealed acetone bath for over 48 hours to dissolve any possible uncured PDMS resin. To confirm the presence of PDMS, the solution was later transferred to a glass container where acetone was evaporated and replaced with CDCl_3_ for NMR. As shown in the figure below, the peak near 4 and 5 ppm can be characterized as the presence of PMDS residue within the acetone solution [1]. To determine any possible post curing, the same experiment was conducted. Two PDMS/acetone solutions were prepared: Sample A was cured in 80^o^C oven for 2 hours while Sample B was cured in 80^o^C for 4 hours. After soaking in equal amounts of acetone for over 24 hours. 1g of each solution was transferred to clean glass jars for weighing. The weight of the jars was first recorded as reference. The final weight of the material can be characterized as the PDMS residue after the acetone fully evaporates, if the post curing assumption is correct. The final weight of Sample A and Sample B should be significantly different, as Sample B is expected to have less uncured residue. Test results shows that Sample A has 8.5 mg of PDMS while Sample B has around 7.8 mg of residue. It is possible that some post curing did occur over prolonged heating but did not contribute significantly to the shape programming ability.


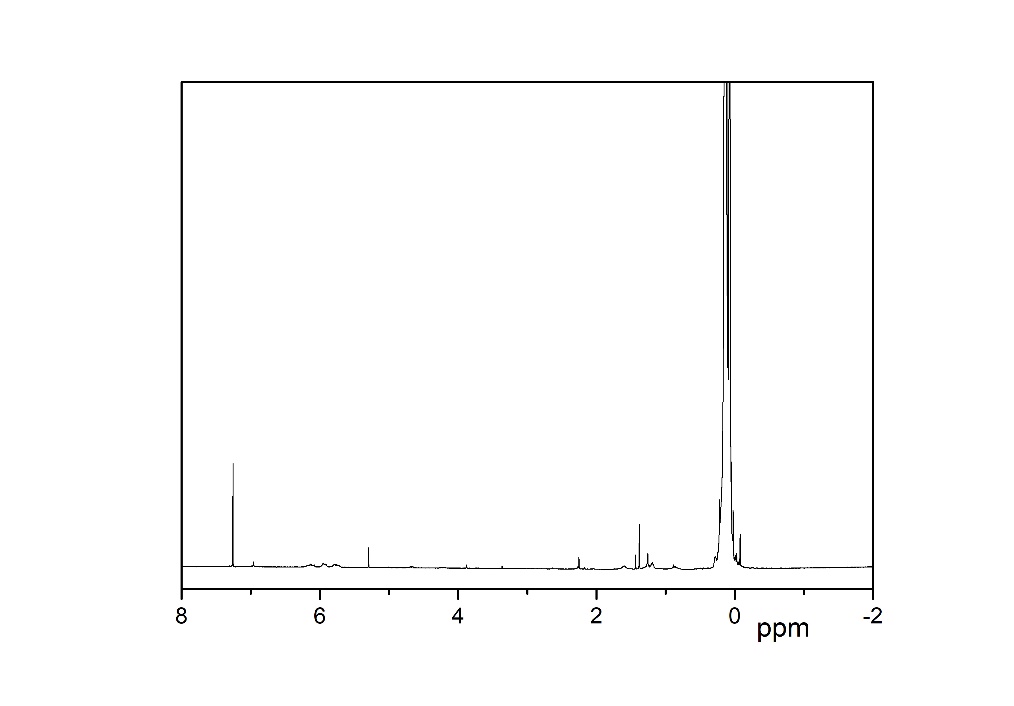


**Figure S4** - NMR spectrum of PDMS

[1] Ramli, M.R., Othman, M.B.H., Arifin, A. and Ahmad, Z., 2011. Cross-link network of polydimethylsiloxane via addition and condensation (RTV) mechanisms. Part I: Synthesis and thermal properties. *Polymer degradation and stability* **96**, 2064-2070 (2011)
